# NR2F1-AS1 Promotes Pancreatic Ductal Adenocarcinoma Progression Through Competing Endogenous RNA Regulatory Network Constructed by Sponging miRNA-146a-5p/miRNA-877-5p

**DOI:** 10.3389/fcell.2021.736980

**Published:** 2021-09-28

**Authors:** Dong Luo, Yunfei Liu, Zhiqiang Li, Hongwei Zhu, Xiao Yu

**Affiliations:** Department of Hepatopancreatobiliary Surgery, Third Xiangya Hospital, Central South University, Changsha, China

**Keywords:** NR2F1-AS1, miR-146a-5p/miR-877-5p, pancreatic ductal adenocarcinoma, ceRNA, bioinformatics analysis

## Abstract

The role of NR2F1-AS1 in pancreatic ductal adenocarcinoma (PDAC) remains unknown. Therefore, we aimed to investigate the biological mechanism of NR2F1-AS1 in PDAC. The expression of NR2F1-AS1 was measured by using microarray data and real-time PCR. The effects of NR2F1-AS1 knockdown on proliferation, cell cycle progression, invasion *in vitro* and tumorigenesis *in vivo* were investigated. The mechanism of competitive endogenous RNAs was determined from bioinformatics analyses and validated by a dual-luciferase reporter gene assay. Potential target mRNAs from TargetScan 7.2 were selected for subsequent bioinformatics analysis. Key target mRNAs were further identified by screening hub genes and coexpressed protein-coding genes (CEGs) of NR2F1-AS1. NR2F1-AS1 was highly expressed in PDAC, and the overexpression of NR2F1-AS1 was associated with overall survival and disease-free survival. The knockdown of NR2F1-AS1 impaired PDAC cell proliferation, migration, invasion and tumorigenesis. NR2F1-AS1 competitively sponged miR-146a-5p and miR-877-5p, and low expression of the two miRNAs was associated with a poor prognosis. An integrative expression and survival analysis of the hub genes and CEGs demonstrated that the NR2F1-AS1–miR-146a-5p/miR-877-5p–GALNT10/ZNF532/SLC39A1/PGK1/LCO3A1/NRP2/LPCAT2/PSMA4 and CLTC ceRNA networks were linked to the prognosis of PDAC. In conclusion, NR2F1-AS1 overexpression was significantly associated with poor prognosis. NR2F1-AS1 functions as an endogenous RNA to construct a novel ceRNA network by competitively binding to miR-146a-5p/miR-877-5p, which may contribute to PDAC pathogenesis and could represent a promising diagnostic biomarker or potential novel therapeutic target in PDAC.

## Introduction

Pancreatic ductal adenocarcinoma (PDAC) remains a highly fatal disease with a 5-year survival rate of 10% (Siegel et al., 2021). PDAC accounts for almost as many deaths (466,000) as cases (496,000) because of its poor prognosis, and it is the seventh leading cause of cancer death in both sexes in 2020 (Sung et al., 2021) and projected to become the second leading cause of cancer-related death by 2030 (Rahib et al., 2014).

Pancreatic ductal adenocarcinoma is characterized by late diagnosis, metastasis and acquired resistance to chemotherapeutic agents in the clinic (Zheng et al., 2015; Sakamoto et al., 2020). Surgical resection with adjuvant systemic chemotherapy currently provides the only chance of long-term survival. However, only10–20% of PDAC patients are diagnosed with localized surgically resectable disease (Strobel et al., 2019). The prognosis for PDAC is still poor despite diagnostic progress and new chemotherapeutic regimens. Therefore, research on the genetic alterations and underlying molecular mechanism of PDAC is still urgently required to find new treatment strategies.

Endogenous genetic alterations, including KRAS oncogenes and mutations or losses of CDKN2A, TP53 and SMAD4, have been well characterized (Biankin et al., 2012; Rishi et al., 2015; Mizrahi et al., 2020). In addition, the critical role of dysregulation of epigenetic modifiers, such as non-coding RNAs, in the development and progression of many human cancer types, including PDAC, is also increasingly emphasized.

Emerging evidence has shown that long non-coding RNAs (lncRNAs) are major regulators in human cancers, including PDAC. LncRNAs are a class of measurably conserved and polyadenylated ncRNAs that are longer than 200 nucleotides, and they do not encode proteins (Lee, 2012; Slack and Chinnaiyan, 2019; Yao et al., 2019). LncRNAs are widely present in organisms and play an important role in various physiological and pathological processes at the level of epigenetic modification, transcription and posttranscriptional regulation.

Several groups have shown that dysregulated expression of lncRNAs is correlated with the initiation, progression, invasion, metastasis, angiogenesis, and drug resistance of PDAC (Lin et al., 2020; Pandya et al., 2020; Zhou et al., 2020). The functions and regulatory mechanisms of lncRNAs in PDAC include potential oncogenes and suppressors (Gong and Jiang, 2020). For lncRNAs as potential oncogenes, a negative correlation was observed in PDAC patients with higher MALAT-1 expression levels and disease-free survival (Liu et al., 2014). HOTAIR was a highly upregulated lncRNA and functioned as an oncogene in PDAC. Interestingly, the overexpression of HOTAIR was reported to have a positive correlation with increased cell growth, survival, migration and invasion in pancreatic carcinoma in a PRC2-dependent and PRC2-independent fashion (Kim et al., 2013; Li et al., 2017). In the context of PDAC, the HOTTIP lncRNA seemed to regulate HOX genes (HOXA9 and HOXA10), and the HOXA9 gene has been shown to promote cancer stem cell proliferation through the Wnt/β-catenin signaling pathway (Fu et al., 2017). On the other hand, overexpression of GAS5 inhibited PDAC cell proliferation, migration and gemcitabine resistance through miR-221/suppressor of cytokine signaling 3 (*SOCS3*)-mediated epithelial-mesenchymal transition (EMT; Liu et al., 2018a). Hu et al. (2016) found that MEG3 inhibits proliferation, induces apoptosis *via* p53 activation and is upregulated along with p53 by fenofibrate to reduce the proliferation of PDAC cells. For the regulatory mechanism, it has been reported that lncRNAs can act as competing endogenous RNAs (ceRNAs) or “RNA sponges,” and they interact with microRNAs in a manner that can sequester these molecules and reduce their regulatory effect on target mRNAs (Cesana et al., 2011; Tay et al., 2014). For example, lncRNA THAP9-AS1 promoted PDAC and led to a poor clinical outcome by sponging miR-484 and interacting with YAP (Li et al., 2020).

The annotated potential lncRNA NR2F1-AS1 (NR2F1 antisense RNA 1) was previously reported to be expressed at low levels in certain human normal tissues, especially in the pancreas (Fagerberg et al., 2014). We found that NR2F1-AS1 was overexpressed in PDAC based on a bioinformatics analysis of publicly available datasets (e.g., Gene Expression Omnibus, GEO). NR2F1-AS1 has been reported to promote the growth of hepatocellular carcinoma (Huang H. et al., 2018), thyroid carcinoma (Yang et al., 2020), osteosarcoma (Li et al., 2019) and breast cancer (Sanchez et al., 2020). However, to the best of our knowledge, the biological role and clinical significance of NR2F1-AS1 and the mechanisms behind these events in PDAC have not been revealed.

The present study aimed to determine NR2F1-AS1 expression in PDAC, investigate the roles of NR2F1-AS1 in PDAC cells and elucidate the potential mechanisms of the ceRNA regulatory network underlying the effect of NR2F-AS1 on PDAC progression by combining experimental and bioinformatics analyses.

## Materials and Methods

### Cell Lines and Culture

The PDAC cell lines PANC-1, CFPAC-1, Capan-2, SW1990, BXPC-3 and an immortalized human pancreatic ductal epithelial cell line (HPDE6) were kindly provided by Dr. Fang He and Hongwei Zhu (Chinese University of Hong Kong, Hong Kong, SAR China). HEK-293Ts were purchased from GENE (Shanghai, China). Cells were cultured in RPMI-1640 or DMEM (Gibco, United States) supplemented with 10% fetal bovine serum. HPDE6 cells were cultured in Keratinocyte-SFM (K-SFM) medium supplemented with Bovine Pituitary Extract (BPF) and Human Recombinant (EGF) [500 ml K-SFM)] medium contain 25 mg BPF and 2.5 μg EGF). Other cells were cultured in DMEM, IMDM, or RPMI 1640 (Gibco, United States) with 10% fetal bovine serum (FBS, Gibco). All cells were cultured at 37°C under 5% CO_2_ in a humidified chamber.

### Quantitative Real-Time PCR

Total RNA was extracted from the PDAC cells using TRIzol reagent (Invitrogen, United States). The RNA concentration and purity were measured at 260/280 nm using a NanoDrop ND-2000 spectrophotometer (Thermo Fisher Scientific, United States). Next, according to the manufacturer’s instructions, cDNA was synthesized with a reverse transcription kit (Toyobo, Osaka, Japan). qRT-PCR was performed on a LightCycler 480 (Roche, Switzerland) using a standard protocol from the SYBR Green PCR Kit (Toyobo, Japan). The qRT-PCR data were normalized to the expression of GAPDH. Primers used for qRT-PCR assays are listed in Supplementary Table 1. For the detection of miRNA expression, reverse transcription was performed and microRNAs were detected with Ploy-A primers purchased from GeneCopoeia (Guangzhou, China). U6 was used as the endogenous control. Relative fold changes were calculated using the comparative delta-delta CT method (2-ΔΔCt). All PCR assays were repeated three times.

### Cell Transfection and Virus Infection

To establish stable transfectants *via* knockdown, shRNA sequences targeting NR2F1-AS1 (target sequence for sh-1#: 5′-GTAGATGAAACTCAAGAGA-3′; sh-2#: 5′-CCACAATATTAACCAGGAT-3′) were designed and inserted in lentiviral plasmids purchased from GENE (Shanghai, China). PANC-1 cells and CFPAC-1 cells were seeded in 6-well plates and infected with lentiviral particles expressing NR2F1-AS1 shRNA following the manufacturer’s instructions to construct stably transfected cell lines. Puromycin (2 μg/ml) was added to the culture medium 72 h after infection and maintained for at least 1 week to select stably transfected cell lines (PANC-1/CFPAC-1-sh NR2F1-AS1 or PANC-1/CFPAC-1-sh-Control).

### Cell Counting Kit-8 Assay

Cell viability was tested using a Cell Counting Kit-8 (CCK-8, Beyotime, Shanghai, China) according to the manufacturer’s instructions and the transfected cells were grown in 96-well plates. Cellular viability was assessed every 24 h (for 96 h) through the measurement of the absorbance at a wavelength of 450 nm by a microplate reader (GEN5, United States).

### Colony Formation Assay

Cells were plated onto 6-well plates (1 × 10^3^ cells/well). The cells were cultured for 14 days and then stained with 0.1% crystal violet, and the number of colonies was counted using ImageJ.

### Wound-Healing Scratch Assay

PANC-1 and CFPAC-1 cells were plated in 6-well plates and cultured in medium containing 10% FBS. After 24 h, the cells grew to almost total confluence. A scratch was created on the monolayer of cells with a 10 μl pipette tip. Subsequently, phosphate-buffered saline was used to wash the cells. Images of the cells that had migrated into the wound were obtained at 0 and 48 h using a microscope (OLYMPUS-IX71, Japan).

### Transwell Invasion and Migration Assay

Approximately 1 × 10^5^ stably transfected PDAC cells (PANC-1 and CFPAC-1) were suspended in serum-free medium and seeded in either chamber (Corning Costar, United States). Chambers not coated with Matrigel were used for migration assays, and chambers coated with Matrigel with 8-μm pores (Corning Costar, United States) were used for invasion assays. For both assays, medium containing 10% FBS was added to the lower chamber as a chemoattractant. After 24 h of incubation, the migrated and invaded cells on the lower membrane surface were fixed with formaldehyde for approximately 20 min. Optical microscopy was applied for cell counting and then stained with 1% purple crystal solution. Five random fields were counted per chamber by using an inverted microscope (OLYMPUS-IX71, Japan). Each experiment was repeated three times.

### Apoptosis Analysis and Cell Cycle Analysis

Cell apoptosis status was determined by following the protocol of the Annexin V-FITC/PI-cell apoptosis Detection Kit (United States). Cells were trypsinized and resuspended in binding buffer containing Annexin V-FITC (United States) and propidium iodide (PI, BD Biosciences) for 20 min in the dark. Stained cells were analyzed using a flow cytometer. For analysis of cell cycle distribution, the cells were fixed with ice-cold 75% ethanol and incubated with PI (50 μg/mL) in the presence of RNase A (Sigma-Aldrich) for 30 min. The DNA content was analyzed by flow cytometry.

### Animal Studies

BALB/C mice (male, 4–6 weeks old) were purchased from the Department of Animals and approved by the animal Ethics Committee of the Third Xiangya Hospital, Central South University. Animals were fed under sterile specific pathogen-free conditions. Each mouse was then injected with 100 μL of stably transfected PDAC (PANC-1 and CFPAC-1) cell suspension (2 × 10^6^cells). Tumor volumes were monitored weekly. After 3 weeks, all mice were sacrificed using cervical dislocation, and tumor weight and volume were measured.

### Luciferase Report Assay

293T cells were seeded onto 24-well plates and transfected with the reporter (NR2F1-AS1-MUT plasmid or NR2F1-AS1-WT) constructs together with the miR-146a-5p/miR-877-5p plasmid or empty vector using X-tremegene HP. The pRL-TK plasmid (Promega, Fitchburg, MA, United States) expressing Renilla luciferase was cotransfected to control for transfection efficiency. Forty-eight hours after transfection luciferase activities were measured using the Dual-Luciferase Reporter Assay System (Promega) according to the manufacturer’s instructions, and the relative luciferase activity was determined after normalization against Renilla luciferase activity.

### Fluorescence *in situ* Hybridization

Cy3-labeled lncRNA NR2F1-AS1 probes were purchased from RuiBo Biomedical Center. PANC-1 cells and CFPAC cells were fixed in 4% formaldehyde and permeabilized with 0.5% Triton X-100. The cells were then hybridized with Cy3-probes. Nuclei were stained with DAPI. Slides were hybridized with probes overnight at 37°C and washed with 4× saline-sodium citrate (4× SSC), 2× SSC and 1× SSC at 42°C for 5 min. Glass slides were mounted using fluorescence mounting medium. Cells were observed and images were acquired using a fluorescence microscope (Olympus).

### Bioinformatics Analysis

Microarray data of NR2F1-AS1 expression were obtained from the GEO dataset (GSE15471) (downloaded at).^1^ The potential microRNA binding sites with NR2F1-AS 1 were predicted by the hTFtarget online^2^ (Zhang Q. et al., 2020). MicroRNA-mRNA binding sites were obtained from TargetScan 7.2 (Agarwal et al., 2015). Database for Annotation, Visualization and Integrated Discovery (DAVID) 6.8 (Huang et al., 2009) was introduced to conduct Gene Ontology (GO) functional annotations and Kyoto Encyclopedia of Genes and Genomes (KEGG) pathway enrichment analyses for potential target mRNA genes. Then, the top 10 enriched GO terms and KEGG pathways were displayed using GraphPad Prism 8. We surveyed cellular signaling pathways involving miRNAs utilizing the miRNACancerMAP database (Tong et al., 2018). The PPI interaction networks between the potential target mRNA genes were constructed by the Search Tool for the Retrieval of Interacting Genes (STRING) database^3^ (Szklarczyk et al., 2019). First, potential target mRNA genes were entered into the database. Then, high-resolution bitmaps were displayed and downloaded from the webpage. Only interactors with a combined confidence score > = 0.4 were shown in the bitmap. The hub genes in the PPI networks were identified using CytoHubba, a plugin in Cytoscape software (Version 3.7.2). According to the node degree, the top 10 hub genes were displayed in Cytoscape. Meanwhile, CEGs of NR2F1-AS1 were determined using a practical and user-friendly web interface called Co-LncRNA, which investigates the lncRNA combinatorial effects in GO annotations and KEGG pathways based on RNA-Seq data. A functional enrichment analysis of CEGs was performed using Metascape Analysis^4^ (Zhou et al., 2019). A gene expression profiling interactive analysis (GEPIA)^5^ was performed to analyze the expression level of NR2F1-AS1 (lncRNA) and key mRNA in PDAC from the pan-cancer analysis (Tang et al., 2017). We obtained a list of upregulated expressed genes in PDAC from a (GEPIA, see text footnote 5). The survival analysis of miRNA expression was based on OncomiR^6^ (Wong et al., 2018). The prognostic values of lncRNAs and key target genes in PDAC were analyzed using the Kaplan-Meier plotter database (Nagy et al., 2018).

### Statistical Analysis

All data are presented as the mean value ± standard deviation (Mean ± SD). The *t*-test and the χ^2^ test were used for comparisons between groups. Statistical analyses were performed using GraphPad Prism 8. Differences were deemed statistically significant at *p* < 0.05.

## Results

### NR2F1-AS1 Is Upregulated in Pancreatic Ductal Adenocarcinoma and Correlated With Poor Prognosis

**FIGURE 1:**
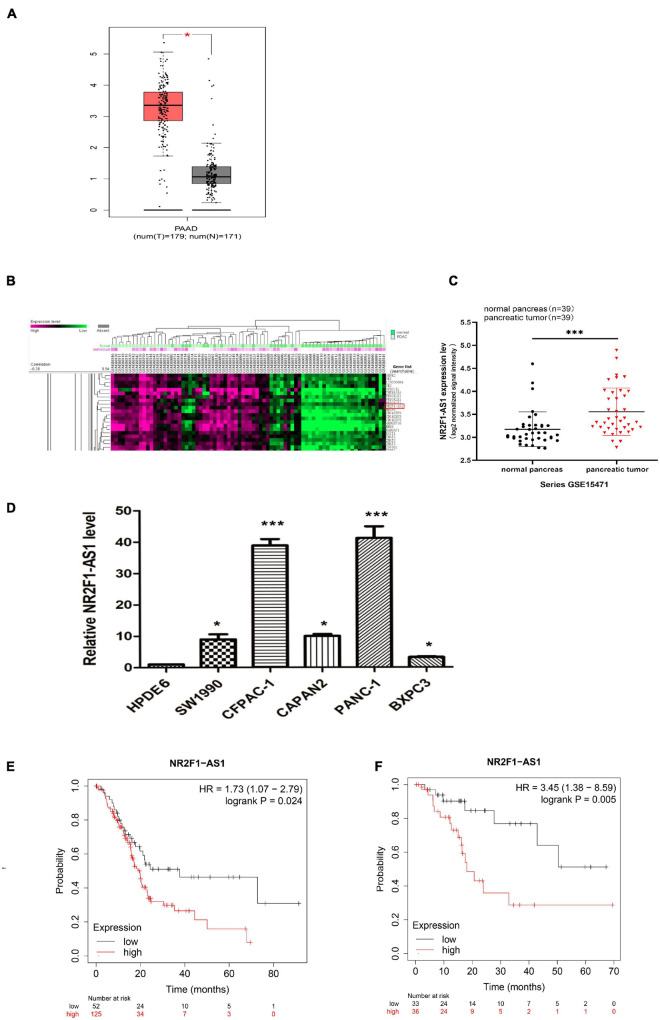
NR2F1-AS1 is upregulated in PDAC and correlated with poor prognosis. **(A)** Boxplots comparing NR2F1-AS1 expression in PDAC samples (*n* = 179) and normal pancreatic tissue samples (*n* = 171). **(B)** Heatmap showing differentially expressed lncRNAs in the GEO dataset of GSE5471. **(C)** Microarray data from the GEO dataset GSE5471 were used to analyze the relative expression of NR2F1-AS1 in PDAC and adjacent normal tissue. **(D)** Relative expression levels of NR2F1-AS1 in PDAC cell lines. **(E,F)** Correlation analysis of NR2F1-AS1 expression with overall survival (OS) and recurrence-free survival (RFS) in PDAC patients obtained from the Kaplan–Meier plotter database. The *P* value was calculated by a log-rank test. HR, hazard ratio. Statistical significance: *p < 0.05 and ***p < 0.001.

To identify eligible lncRNAs in PDAC, we queried public online datasets. We downloaded a total of 4711 lncRNAs (Supplementary Table 2), with valid names obtained from the HGNC database of human gene names when this work was initiated, they were analyzed by GEPIA. We were particularly interested in NR2F1-AS1 because its relative expression was the highest among the PDAC tumor samples relative to adjacent normal tissues in the pan-cancer analysis (Supplementary Figure 1), and this upregulation was confirmed (Figure 1A). A publicly accessible microarray dataset (GEO:GSE15471) from PDAC patients was analyzed, and the result was consistent with data from the GEPIA. We found that NR2F1-AS1 was significantly increased in the PDAC samples relative to the normal samples (Figures 1B,C). Moreover, NR2F1-AS1 expression in PDAC cell lines was also increased compared with HPDE6 based on RT-qPCR, especially in the PANC-1 and CFPAC-1 cell lines (Figure 1D). To clarify the prognostic value of NR2F1-AS1 among PDAC patients, the relationship between its expression and survival time was investigated by Kaplan–Meier plotter. This result indicated that PDAC patients with higher NR2F1-AS1 expression had shorter OS (*p* = 0.024) and RFS (*p* = 0.005) (Figures 1E,F). Collectively, these findings indicated that upregulated expression of NR2F1-AS1 might be closely associated with poor outcomes of PDAC. Therefore, we focused on NR2F1-AS1 for further characterization.

### Knockdown of NR2F1-AS1 Suppresses Pancreatic Ductal Adenocarcinoma Growth *in vitro* and *in vivo*

**FIGURE 2:**
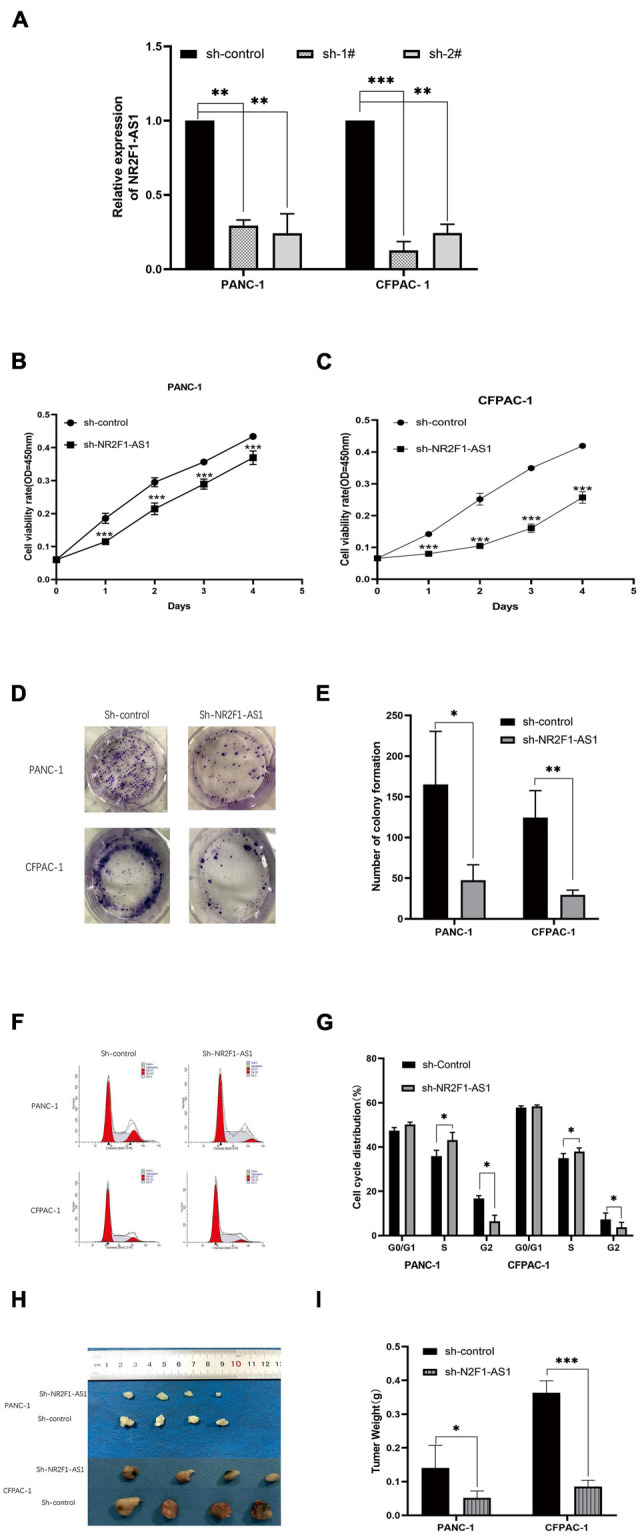
Downregulation of NR2F1-AS1 expression suppresses PDAC cell proliferation and tumor growth *in vitro* and *in vivo*. **(A)** Expression of NR2F1-AS1 in PANC-1 and CFPAC-1 cells transfected with NR2F1-AS1 shRNA. **(B,C)** Knockdown of NR2F1-AS1 significantly suppressed proliferation in PANC-1 and CFPAC cells compared with the control group. Cell viability was determined by CCK-8 assay. **(D,E)** Colony forming assays after sh-NR2F1-AS1 transfection in PANC-1 and CFPAC-1 cells. **(F,G)** Flow cytometry was carried out to examine the cell cycle status of PANC-1 and CFPAC-1 cells after transfection with either sh-NR2F1-AS1 or sh-control. **(H,I)** Knockdown of NR2F1-AS1T reduces the tumor weight. The nude mice were sacrificed 21 days after the injection, and tumors from the respective groups are shown. **(H)** Image of the tumors; **(I)** tumor weight. ^∗^*p* < 0.05, ^∗∗^*p* < 0.01, and ^∗∗∗^*p* < 0.001.

To determine the biological function of NR2F1-AS1 in PDAC, loss-of function approaches were employed. PANC-1 and CFPAC-1 lines were selected to further investigate its effects in PDAC as described above (Figure 1E). First, NR2F1-AS1 was knocked down using shRNAs. Then, stable NR2F1-AS1 knockdown in the PANC-1 and CFPAC-1 lines expressing sh-1# or sh-2# (shRNAs) was established. NR2F1-AS1 expression was suppressed by shRNA transfection in the PANC-1 and CFPAC-1 cell lines (Figure 2A). Loss of NR2F1-AS1 significantly suppressed the proliferation of PANC-1 and CFPAC-1 cells (Figures 2B,C), as determined by CCK-8 assay. The colony-forming assay also showed that NR2F1-AS1 knockdown significantly inhibited the colony-forming capacity of PDAC cells (Figures 2D,E). Next, we performed flow cytometry to examine whether NR2F1-AS1 could affect the proliferation of PDAC cells by altering cell cycle progression. The results showed that the cell cycle progression of sh-NR2F1-AS1 cells was arrested in S phase and the percentage of G2 phase cells was decreased compared with that of cells transfected with the sh-control (Figures 2F,G). However, the statistics showed no significant difference, downregulation of NR2F1-AS1 seemed to promote cell apoptosis (Supplementary Figure 2). To further test whether the level of NR2F1-AS1 expression could affect PDAC cell growth *in vivo*, NR2F1-AS1 stable knockdown cells and control cells were subcutaneously injected into BALB/C nude mice. Twenty-one days after the cells were injected, the mice were euthanized, and tumors were measured (Figure 2H). The tumor weight at the end of the experiment was markedly lower in the sh-NR2F1-AS1-transfected PANC-1 and CFPAC-1 groups than in the control group (Figure 2I). These results suggested that NR2F1-AS1 is a functionally important lncRNA in PDAC.

### Knockout of NR2F1-AS1 Abrogates Migration and Invasion Ability in Pancreatic Ductal Adenocarcinoma

**FIGURE 3:**
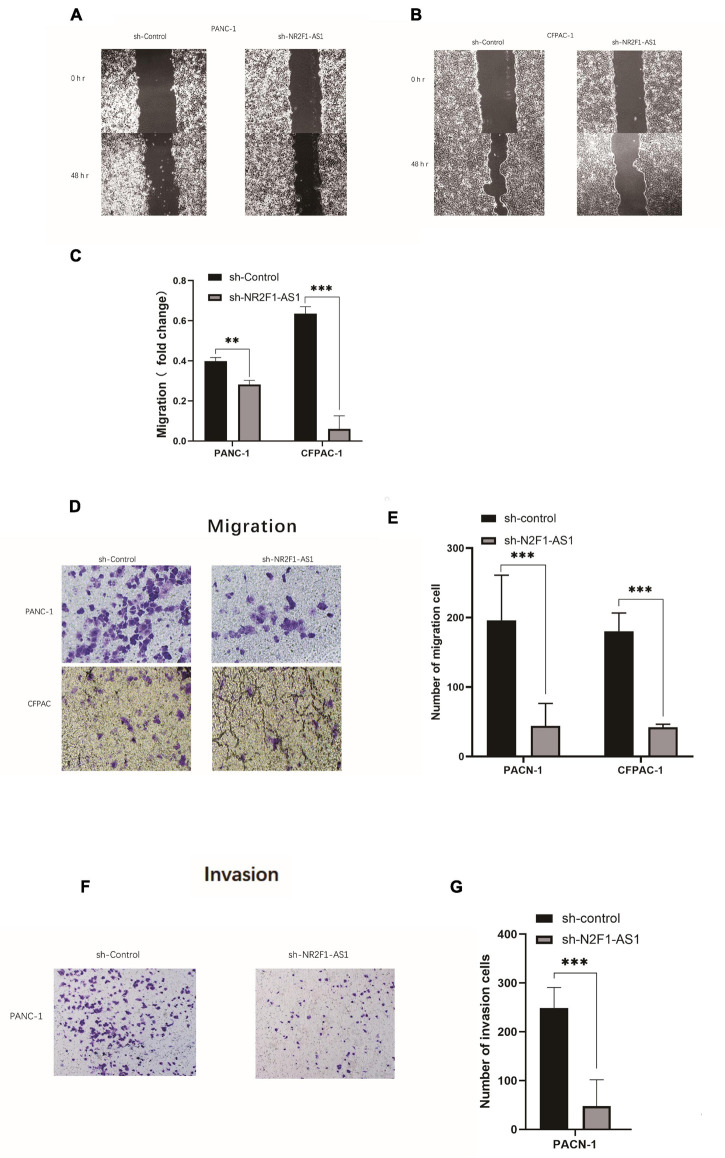
Knockout of NR2F1-AS1 abrogates migration and invasion ability in PDAC. The migration and invasion abilities of tumor cells were assessed using wound healing assays as well as Transwell migration and invasion assays. **(A,B)** Wound healing assays (photographed after 0 and 48 h). **(C)** Histograms showing the change in healing area. **(D)** Transwell migration assays (photographed after 24 h). **(E)** Histograms showing the numbers of cells that migrated after 24 h. **(F)** Transwell invasion assays (photographed after 24 h). **(G)** Histograms showing the numbers of cells that invaded after 24 h. Statistical significance: **p < 0.01 and ***p < 0.001.

Pancreatic ductal adenocarcinoma is characterized by early metastasis or aggressive tumor spread. Given that high expression of NR2F1-AS1 was significantly associated with poor OS and DFS (Figures 1E,F), we hypothesized that NR2F1-AS1 expression is critical for cancer cell migration and invasion. We then assessed tumor cell migration and invasion abilities using wound-healing assays as well as Transwell migration and invasion assays. Knockdown of NR2F1-AS1 in PANC-1 or CFPAC-1 cells markedly suppressed cell migration and invasion compared with the corresponding control cells (Figures 3A–G).

### NR2F1-AS1 Acts as a Sponge for miR-146a-5p/miR-877-5p in Pancreatic Ductal Adenocarcinoma

**FIGURE 4:**
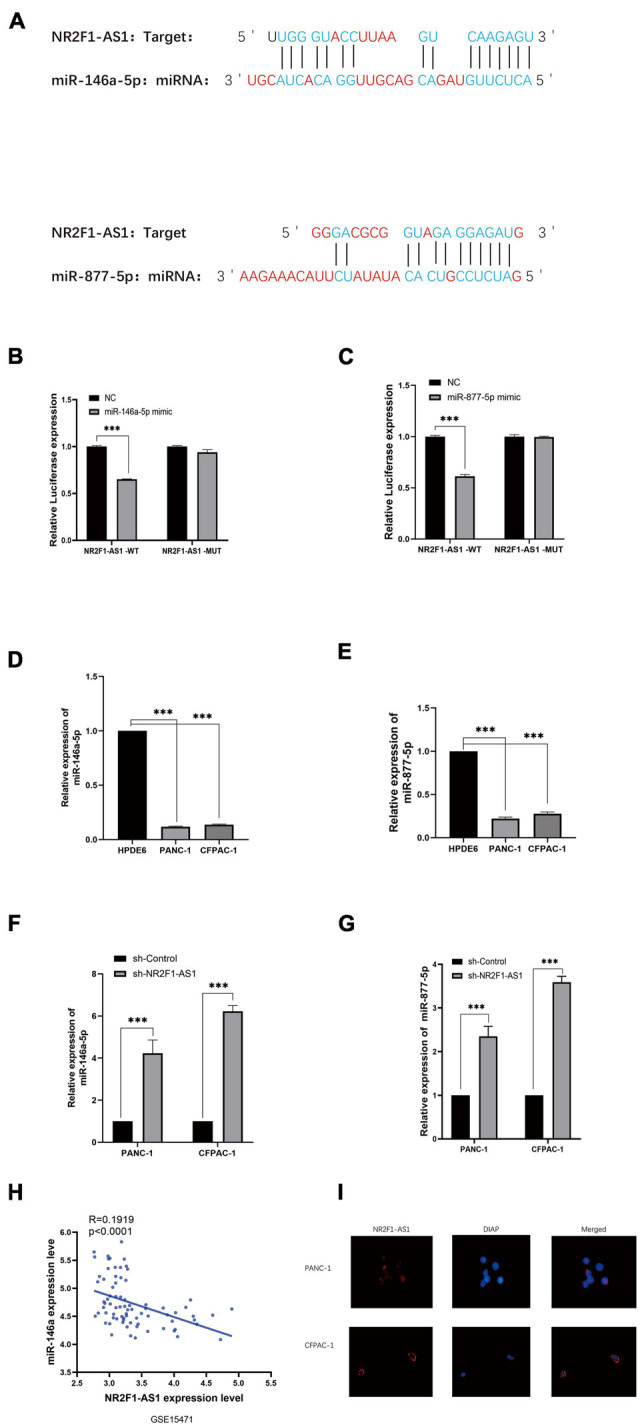
NR2F1-AS1 interacts with miR-146a-5p/miR-877-5p in PDAC. **(A)** Prediction of target sites for miR-146a-5p/miR-877-5p in NR2F1-AS1 based on the online program. **(B,C)** Luciferase reporter assay showed that miR-146a-5p/miR-877-5p overexpression significantly suppressed the activity of the reporter containing wild-type NR2F1-AS1. **(D,E)** Expression levels of miR-146a-5p and miR-877-5p in PANC-1 and CFPAC-1 cells. **(F,G)** RT-PCR was performed with sh-NR2F1-AS1-transfected PANC-1 and CFPAC-1 cells to determine the effects of NR2F1-AS1 on miR-146a-5p and miR-877-5p. **(H)** Expression of NR2F1-AS1 was negatively associated with the expression of miR-146a-5p in 39 PDAC tissues from GSE15471. **(I)** FISH images showing the localization of NR2F1-AS1 in PANC-1 and CFPAC-1 cells. Statistical significance: ***p < 0.001.

To explore the mechanism by which NR2F1-AS1 affects PDAC growth, we performed a bioinformatics analysis to search for miRNAs that can interact with NR2F1-AS1. After screening for miRNAs using the hTFtarget online tool. The results showed that NR2F1-AS1 harbors putative target sites for 40miRNAs (Supplementary Table 3), especially miR-146a-5p/miR-877-5p. (Figure 4A). To determine the direct binding between NR2F1-AS1 and miR-146a-5p/miR-877-5p, dual-luciferase reporter assays were carried out. NR2F1-AS1-wt or NR2F1-AS1-mut was cotransfected into HEK293T cells with miR-146a-5p/miR-877-5p mimics or the negative control. The results revealed that miR-146a-5p/miR-877-5p overexpression considerably reduced the luciferase activity of the NR2F1-AS1-wt luciferase reporter vector compared with the negative control, while miR-146a-5p/miR-877-5p overexpression did not have any impact on the luciferase activity of NR2F1-AS1-mut (Figures 4B,C). RT-qPCR was then performed to determine the expression of miR-146a-5p and miR-877-5p among the PANC-1 and CFPAC-1 cell lines. The findings indicated that compared with HPDE6, the relative expression of miR-146a-5p and miR-877-5p was markedly decreased (Figures 4D,E). The interaction between NR2F1-AS1 and miR-146a-5p/miR-877-5p was further investigated, and knockdown of NR2F1-AS1 expression markedly increased miR-146a-5p/miR-877-5p expression in both cell lines (Figures 4F,G). Furthermore, we found that the expression of NR2F1-AS1 was negatively associated with the expression of miR-146a-5p in 39 PDAC tissues from GSE15471 (Figure 4H). Finally, we performed a FISH analysis to identify the distribution of NR2F1-AS1 in PDAC cells. The results showed that NR2F1-AS1 was abundant in both the cytoplasm and nucleus and mainly located in the cytoplasm of PDAC cells (Figure 4I). These results suggested that NR2F1-AS1 directly targeted and negatively regulated miR-146a-5p/miR-877-5p in PDAC.

### Prognostic Values of miR146a-5p/miR-877-5p and Signaling Pathways That May Be Involved in Downstream Targets in Pancreatic Ductal Adenocarcinoma

**FIGURE 5:**
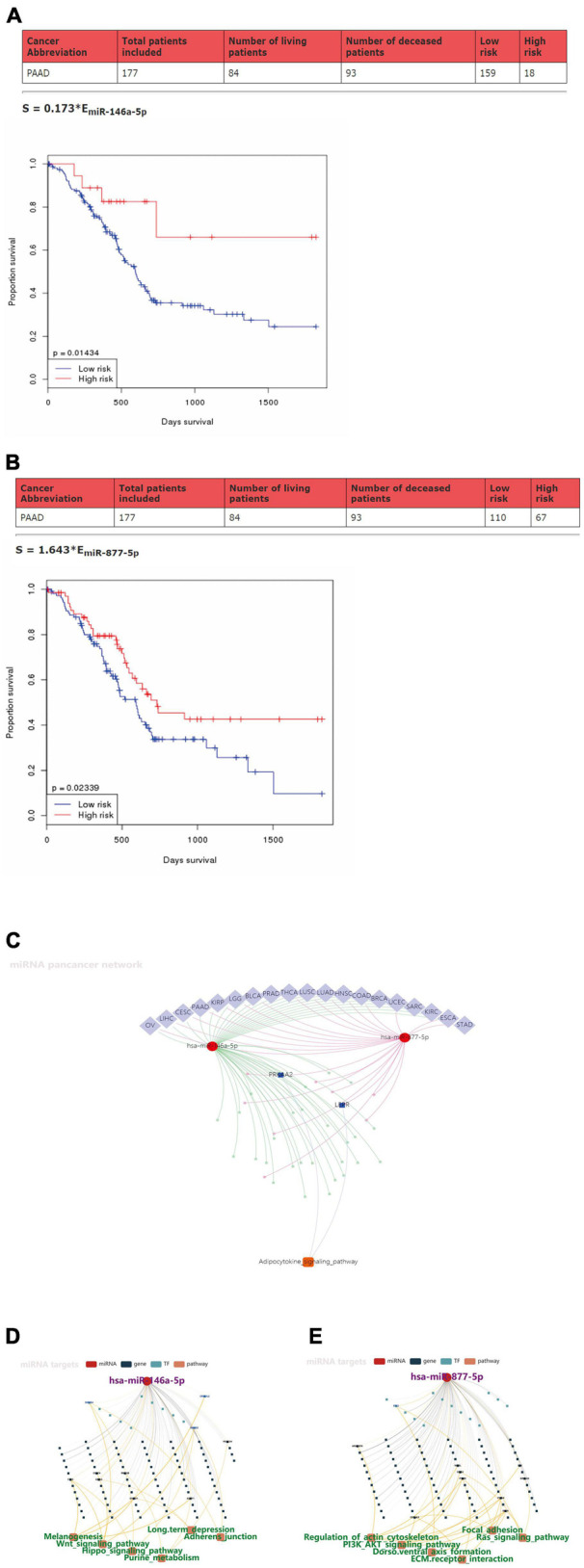
Prognostic values of miR-146a-5p/miR-877-5p and the signaling pathways that may be involved in downstream targets in PDAC. **(A,B)** Kaplan–Meier survival analysis of miR146a-5p and miR-877-5p expression in pancreatic cancer based on OncomiR datasets. **(C)** Pan-cancer microRNA-gene-pathway network from miRNACancerMAP. **(D,E)** Regulatory networks of miR146a-5p/miR-877-5p and associated signaling pathways of putative targets in PDAC from miRNACancerMAP.

To further explore the clinical value of miR-146a-5p/miR-877-5p, we performed an analysis of the relationship between the expression of miR-146a-5p and miR-877-5p and clinicopathological features from OncomiR datasets. A survival analysis based on this database showed that low miR-146a-5p and miR-877-5p expression was associated with poor prognosis in PDAC patients (Figures 5A,B). Then, we surveyed cellular signaling pathways involving miR146a-5p and miR-877-5p by utilizing the miRNACancerMAP database. The results showed that these two miRNAs were involved in multiple tumors, including PDAC (Figure 5C). The results also showed that these two miRNAs with signaling cascades were closely related to tumor growth in PDAC. For miR146a-5p, its activity was associated with melanogenesis, the Wnt signaling pathway, the Hippo signaling pathway, purine metabolism, long-term depression and adherens junctions (Figure 5D). For miR-877-5p, its activity was associated with regulation of the actin cytoskeleton, PI3K-AKT signaling pathway, Dorso ventral axis formation, ECM receptor interaction, focal adhesion and Ras signaling pathway (Figure 5E).

### Preliminary Construction of a Novel lncRNA-miRNA-mRNA (ceRNA) Regulatory Network

**FIGURE 6:**
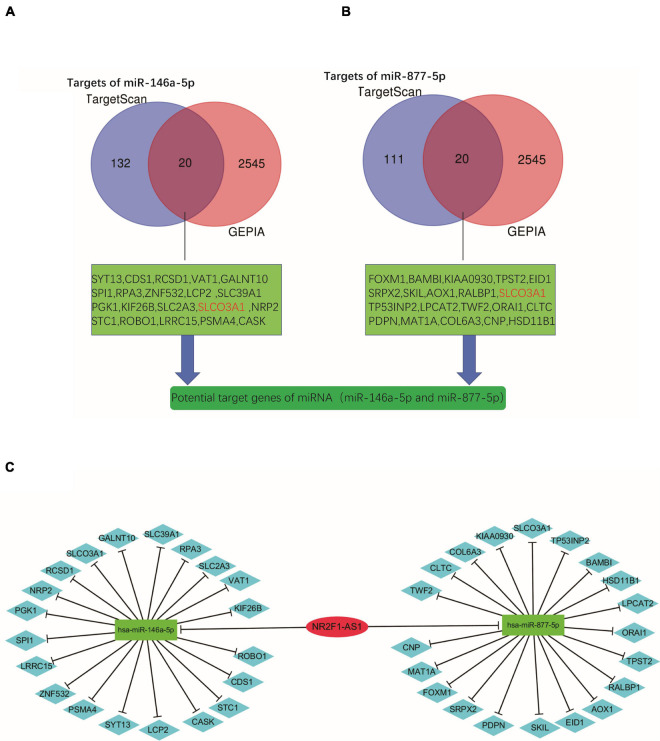
Preliminary construction of a novel lncRNA-miRNA-mRNA (ceRNA) regulatory network. **(A,B)** Venn analysis for preliminary identification of potential target genes by combining prediction analyses and expression using TargetScan7.2 and GEPIA databases. **(C)** Construction of the lncRNA-miRNA-gene network using Cytoscape software.

Subsequently, we predicted downstream target genes of miRNAs (miR-146a-5p and miR-877-5p) using TargetScan7.2. We obtained 152 and 131 potential target mRNA genes for miR-146a-5p and miR-877-5p, respectively (Supplementary Tables 4, 5). Based on the classical inverse relationship between miRNA and the target gene and considering that miR-146a-5p and miR-877-5p were downregulated in PDAC, we hypothesized that the downstream target mRNAs of the two miRNAs should be upregulated. Next, we identified a total of 2565 significantly upregulated DEGs in PDAC from GEPIA (Supplementary Table 6) and further identified significant DEGs that were consistent between the two datasets (TargetScan 7.2 and GEPIA). A total of 39 significantly upregulated potential target genes for miR-146a-5p and miR-877-5p in PDAC were identified (Figures 6A,B and Supplementary Table 6). Finally, we found that miR-146a-5p and miR-877-5p could potentially regulate the expression of 39 key genes (Supplementary Table 7) and constructed a ceRNA regulatory network using Cytoscape software (Figure 6C).

### Identification of Potential Key mRNA Genes in Pancreatic Ductal Adenocarcinoma by Hub Genes and Co-LncRNA Analysis

**FIGURE 7:**
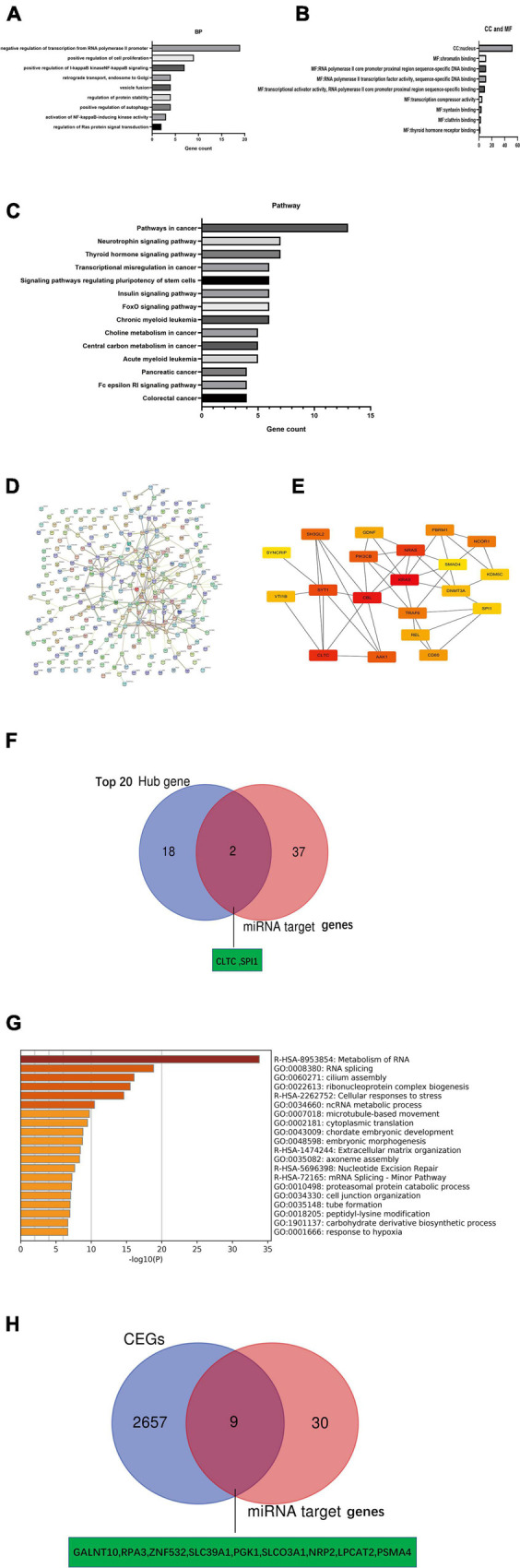
Identification of key mRNA genes in PDAC by hub gene screening and Co-lncRNA analysis. **(A–C)** GO functional annotation and KEGG pathway enrichment analysis for the potential target genes of miRNAs by TargetScan 7.2. **(D,E)** Top 20 hub genes identified in protein-protein interaction (PPI) networks. **(F)** Intersection of the top 20 hub genes and 39 miRNA target genes by Venn analysis. **(G)** Bar graph of enriched terms across input gene lists [2,666 coexpressed protein-coding genes (CEGs) of NR2F1-AS1], colored by *p*-values from Metascape. **(H)** Intersection of 2666 coexpressed protein-coding genes (CEGs) of NR2F1-AS1 from Co-lncRNA and 39 miRNA target genes by Venn analysis.

We obtained 152 and 131 potential target genes for miR-146a-5p and miR-877-5p, respectively, (Supplementary Tables 4, 5), from TargetScan 7.2 as described above. To explore the underlying biological function and corresponding pathways of the potential target genes, the DAVID database was introduced to perform a functional enrichment analysis, which included three GO terms (BP: biological process; CC: cellular component; MF: molecular function) and KEGG pathways. The enriched GO functions included negative regulation of transcription from the RNA polymerase II promoter, positive regulation of cell proliferation and positive regulation of I-kappaB kinase NF-kappaB signaling in the BP category; Chromatin binding, RNA polymerase II core promoter proximal region sequence-specific DNA binding, RNA polymerase II transcription factor activity and sequence-specific DNA binding were identified for the MF category and nucleus was identified in the CC category (Figures 7A,B). The KEGG pathway enrichment analysis revealed that these potential target genes were significantly enriched in some cancer-associated pathways, such as the neurotrophin signaling pathway and pathways regulating stem cell pluripotency, pancreatic cancer, colorectal cancer and small cell lung cancer (Figure 7C). Based on the STRING database analysis, the PPI networks of these potential target genes were constructed (Figure 7D). Then, we identified the top 20 hub genes using Cytoscape software (Figure 7E). Subsequently, we identified 2 key potential target genes (CLTC and SPI1) by Venn analysis of the top 20 hub genes and 39 potential miRNA target genes (Figure 7F). Meanwhile, we identified CEGs of NR2F1-AS1 by using ColncRNA and downloaded a total of 2666 CEGs from Co-LncRNA (Supplementary Table 8). Subsequently, those 2666 CEGs were submitted into the online dataset Metascape for Gene Annotation & Enrichment Analysis. The results of the functional enrichment analysis mainly focused on metabolism of RNA, RNA splicing, cilium assembly, ribonucleoprotein complex biogenesis, cellular responses to stress and ncRNA metabolic processes (Figure 7G). Similarly, 9 key potential target genes (GALNT10, RPA3, ZNF532, SLC39A1, PKG1, SLCO3A1, NRP2, LPCAT2, and PSMA4) were identified by the Venn analysis of 2666 CEGs and 39 potential miRNA target genes (Figure 7H). Finally, we obtained 11 potential key target genes (Supplementary Table 9).

### Further Validation of Key Target Genes With Prognosis in Pancreatic Ductal Adenocarcinoma

**FIGURE 8:**
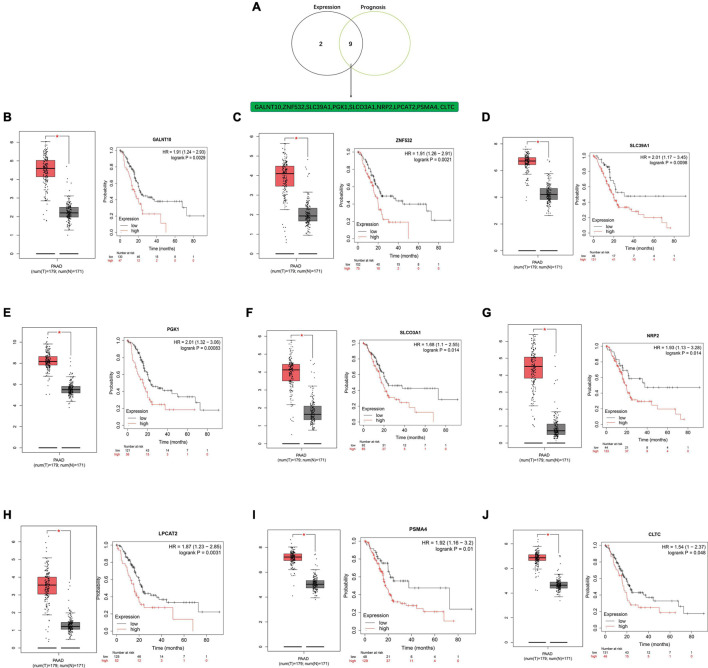
Screening key mRNA (target genes) in PDAC. **(A)** Identification of key mRNAs among the 11 potential key mRNAs by combining expression and prognosis analyses using the GEPIA and Kaplan-Meier plotter databases. **(B)** Expression and prognostic value of GALNT10 in PDAC. **(C)** Expression and prognostic value of ZNF532 in PDAC. **(D)** Expression and prognostic value of SLC39A1 in PDAC. **(E)** Expression and prognostic value of PGK1 in PDAC. **(F)** Expression and prognostic value of SLCO3A1 in PDAC. **(G)** Expression and prognostic value of NRP2 in PDAC. **(H)** Expression and prognostic value of LPCAT2 in PDAC. **(I)** Expression and prognostic value of PSMA4 in PDAC. **(J)** Expression and prognostic value of CLTC in PDAC.

A negative correlation is observed between miRNA and mRNA based on the ceRNA hypothesis. Thus, we analyzed the expression of these mRNAs in PDAC from the GEPIA database. All 11 potential key mRNAs were significantly upregulated in PDAC samples compared with normal samples (Figure 8). A subsequent survival analysis for the 11 potential key mRNA genes demonstrated that patients with high expression of GALNT10, ZNF532, SLC39A1, PGK1, SLCO3A1, NRP2, LPCAT2, PSMA4, and CLTC had unfavorable prognosis. A combined expression and survival analysis for these potential key mRNAs further confirmed 9 key mRNAs (GALNT10, ZNF532, SLC39A1, PGK1, SLCO3A1, NRP2, LPCAT2, PSMA4, and CLTC) (Figure 8).

### Construction of a Novel lncRNA-miRNA-mRNA Network (NR2F1-AS1—miR-146a-5p/miR-877-5p-mRNA) in Pancreatic Ductal Adenocarcinoma

**FIGURE 9:**
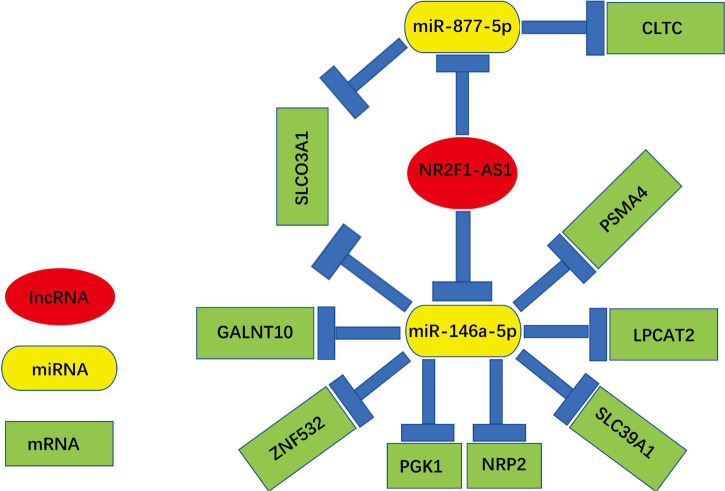
Novel key lncRNA-miRNA-mRNA network competing endogenous RNA (ceRNA) regulatory network associated with the prognosis of PDAC.

A novel triple regulatory network (lncRNA-miRNA-mRNA) with competitive endogenous RNA in PDAC was constructed by combining experimental and bioinformatics analyses. The network totally contained 2 lncRNA-miRNA pairs (NR2F1-AS1—miR-146a-5p and NR2F1-AS1—miR-877-5p), 10 miRNA-mRNA pairs (miR-146a-5p—GALNT10, miR-146a-5p—ZNF532, miR-146a-5p—PGK1, miR-146a-5p—NRP2, miR-146a-5p—SLC39A, miR-146a-5p—LPCAT2, miR-146a-5p—PSMA4, miR-146a-5p—SLCO3A1, miR-877-5p—SLCO3A1, and miR-877-5p—CLTC) and 9 lncRNA-mRNA pairs (NR2F1-AS1—GALNT10, NR2F1-AS1—ZNF532, NR2F1-AS1—PGK1, NR2F1-AS1—NRP2, NR2F1-AS1—SLC39A, NR2F1-AS1—LPCAT2, NR2F1-AS1—PSMA4, NR2F1-AS1—SLCO3A1, and NR2F1-AS1—CLTC). This network is shown in Figure 9. We constructed a novel NR2F1-AS1—miR-146a-5p/miR-877-5p-mRNA network that was significantly associated with the prognosis of PDAC. We believe that NR2F1-AS1 may be a promising diagnostic biomarker or potential novel therapeutic target for PDAC.

## Discussion

The discovery of various lncRNAs in humans has dramatically changed our understanding of the mechanism of cancer. Emerging evidence has shown the important roles of lncRNAs in cancers, such as in the proliferation (Lin et al., 2020), metabolism (Tang et al., 2019), metastasis (Pan et al., 2020), EMT (Liang et al., 2018) and cell stemness of cancer (Zhan et al., 2020). Numerous lncRNAs are dysregulated in PDAC, and this aberration plays a key role in PDAC initiation and progression by regulating a variety of cancer-related biological events (Deng et al., 2018; Huang C.S. et al., 2018; Zeng et al., 2019; Pandya et al., 2020). Recently, Harvard Medical School proposed the hypothesis that competing endogenous RNA (ceRNA) regulates gene expression (Salmena et al., 2011). The ceRNA mechanism hypothesis proposed that these RNA transcripts act as ceRNAs or natural microRNA sponges. Therefore, lncRNAs act as molecular sponges to regulate the levels of mRNAs by competitively binding their same miRNAs targeting mRNAs (Fu et al., 2017; Thomson and Dinger, 2016; Zhong et al., 2018). This hypothesis was later confirmed (Tay et al., 2011; Karreth et al., 2015; Wang et al., 2016; Lin et al., 2020). Emerging evidence has demonstrated that endogenous lncRNAs may participate in posttranscriptional regulation by functioning as ceRNAs in PDAC (Cesana et al., 2011; Lei et al., 2019; Shi et al., 2019).

Initially, we predicted that NR2F1-AS1 was upregulated in PDAC based on public datasets, and the overexpression was further confirmed by a GEO dataset and PDAC cell lines. The upregulated expression of NR2F1-AS1 was associated with poor clinical outcomes by the bioinformatics analysis. After NR2F1-AS1 was knocked down, PDAC cell proliferation, colony formation, migration and invasion were all repressed. In addition, the knockdown of NR2F1-AS1 arrested PANC-1 and CFPAC-1 cells at the G2/M phase. Moreover, NR2F1-AS1 knockdown inhibited PDAC tumor growth *in vivo*. NR2F1-AS1 was also up-regulated in oxaliplatin-resistant hepatocellular carcinoma tissue and cells and NR2F1-AS1 knockdown reduced the invasion, migration in cells (Huang H. et al., 2018). These data validated the important role for NR2F1-AS1 in PDAC growth, thus highlighting the importance of NR2F1-AS1 as a PDAC promoter.

Bioinformatics prediction tools are an emerging assistant method to help researchers discover the underlying molecular mechanism within lncRNAs and miRNAs/mRNAs (Zhang and Yang, 2017). We first performed a bioinformatics analysis to predict potential lncRNA-miRNA interactions using the online tool hTFtarget. The results showed that NR2F1-AS1 harbors putative target sites for miR-146a-5p/miR-877-5p, which was confirmed through dual-luciferase reporter gene assays.

Mechanistically, NR2F1-AS1 can be negatively regulated by miR-146a-5p/miR-877-5p. We found that the expression of miR-146a-5p and miR-877-5p was markedly decreased in PDAC cells while knockdown of NR2F1-AS1 expression markedly increased miR-146a-5p/miR-877-5p expression in both cell lines. For example, we found that the expression of NR2F1-AS1 was negatively associated with the expression of miR-146a-5p in 39 PDAC tissues from GSE15471. Finally, the FISH results revealed that NR2F1-AS1 was mainly expressed in the cytoplasm. Study showed that NR2F1-AS1 is mainly located in the cytoplasm of osteosarcoma (OS) cells and plays an oncogenic role in OS through sponging miR-483-3p (Li et al., 2019). Taken together, NR2F1-AS1 confers an aggressive phenotype by sponging miR-146a-5p/miR-877-5p.

Previous studies have revealed that miR-146a-5p plays a tumor suppressive role in many cancers, such as leukemia (Su et al., 2020), cervical cancer (Dong et al., 2019), lung cancer (Mohamed et al., 2019), gastric cancer (Adami et al., 2019), and breast cancer (Long et al., 2019). Moreover, it has been previously reported that the expression of miR-146a-5p is downregulated in PDAC and acts as a tumor suppressor (Li et al., 2010; Meng et al., 2020). miR-877-5p has also been confirmed as a tumor suppressor in cancer, such as in hepatocellular carcinoma (Yan et al., 2018), gastric cancer (Wu et al., 2020) and cervical cancer (Liang J. et al., 2020). However, little is known about its function or link to NR2F1-AS1 in PDAC.

To the best of our knowledge, we are the first to demonstrate that NR2F1-AS1 acts as a ceRNA by sponging miR-146a-5p/miR-877-5p to regulate the development of PDAC. Subsequently, we found that down-regulated expression of miR-146a-5p and miR-877-5p was associated with poor prognosis in PDAC patients. In addition, miR-146a-5p and miR-877-5p may be involved in multiple tumors, including PDAC, and this result was consistent with the above discussion. The potential target genes of miR-146a-5p and miR-877-5p revealed their participation in several signaling cascades related to tumor development (e.g., Wnt signaling pathway, Hippo signaling pathway and Ras signaling pathway). Combining the potential target mRNAs for miR146a-5p and miR-877-5p in the TargetScan 7.2 database with data from GEPIA, we obtained 39 potential target genes and then preliminarily constructed a novel lncRNA-miRNA-mRNA (ceRNA) regulatory network.

We further identified key target mRNAs by screening hub genes and CEGs of NR2F1-AS1. On the one hand, enrichment analyses of target genes revealed their participation in some GO terms that were associated with cancer biological behaviors, including negative regulation of transcription from the RNA polymerase II promoter (Steinbach et al., 2019), chromatin binding (Heinonen et al., 2015)and nucleus (Kim et al., 2018). The KEGG pathway analysis showed that these target genes were mainly enriched in some cancer-associated pathways, such as pathways in cancer and signaling pathways regulating the pluripotency of stem cells and pancreatic cancer. A PPI network based on these genes was next constructed to obtain the top 20 hub genes and 2 potential key target mRNAs (CLTC and SPI1) were identified by Venn analysis between the top 20 hub genes and 39 potential miRNA target genes. On the other hand, 2,666 CEGs of NR2F1-AS1 were obtained, and the results of the functional enrichment analysis among these CEGs revealed that they were mainly focused on RNA metabolism, RNA splicing, ribonucleoprotein complex biogenesis, cellular responses to stress and ncRNA metabolic processes. Similarly, 9 potential key target genes were identified by the Venn analysis between the 2,666 CEGs and 39 potential miRNA target genes. Therefore, a total of 11 potential key target mRNAs were selected for further expression verification and survival analysis. Finally, a total of 9 key target genes (GALNT10, ZNF532, SLC39A1, PGK1, SLCO3A1, NRP2, LPCAT2, PSMA4, and CLTC) with poor prognosis were defined as key mRNAs in PDAC.

Intriguingly, these 9 key genes have been well investigated in multiple cancers (including PDAC). High GALNT10 expression confers an immunosuppressive microenvironment, promotes tumor progression, predicts poor clinical outcomes in high-grade ovarian serous cancer (HGSC; Zhang G. et al., 2020) and promotes proliferation and apoptosis resistance of hepatoma cells (Wu et al., 2015). ZNF532 drives dramatic mistargeting of active chromatin in NUT midline carcinoma (NMC; Alekseyenko et al., 2017). SLC39A1, one of the Zn^2+^ transporters of SLC families 39, is involved in specific functions in the pancreas, such as insulin synthesis and secretion and metallation of digestive proenzymes. Defective or dysregulated Zn^2+^ metabolism in the pancreas is associated with cancer (Schweigel-Rontgen, 2014). PGK1 regulates metabolism (glycolysis), promotes cell proliferation in brain tumors (Qian et al., 2019) and preferentially supports proliferation by functioning as a glycolytic enzyme in PDAC (Liang C. et al., 2020). SLCO3A1 could potentially be used to target anticancer drugs to PDAC (Hays et al., 2013). High expression of NRP2 is associated with poor overall survival for PDAC (Liu et al., 2018b) and hepatocellular carcinoma patients (Dong et al., 2017). Increased expression of LPCAT2 is associated with poor prognosis of PDAC patients (Idichi et al., 2018) and positively correlated with aggressive prostate cancer (Williams et al., 2014). PSMA4 plays a direct role in cell proliferation in lung carcinoma cell lines (Liu et al., 2009). CLTC promoted tumorigenesis in hepatocellular carcinoma (Huang et al., 2017) and cell growth in breast cancers (Ujihira et al., 2015). The above research can support the accuracy of our bioinformatics analyses.

Therefore, a prognosis-associated lncRNA-miRNA-mRNA network in PDAC was successfully established. Although highly interesting findings were obtained in a series of lab experiments and bioinformatics analyses in this study, more lab experiments need to be performed in the future.

## Conclusion

In conclusion, this study suggests that NR2F1-AS1 is an independent prognostic factor in PDAC patients and promotes proliferation, migration and invasion in PDAC both *in vitro* and *in vivo*; moreover, a novel NR2F1-AS1-miR146a-5p/miR-877-5p-mRNA ceRNA regulatory network was constructed by integrated lab experiments and bioinformatics analysis, and all the RNAs in the network possess significant predictive value for prognosis in PDAC. In addition to identifying the prognostic value of this lncRNA-miRNA-mRNA network in PDAC, this study provided key insights for investigating the molecular mechanism in PDAC. However, additional studies should be conducted to further validate these findings.

## Data Availability Statement

The raw data that support the conclusion of this article will be made available by the authors, without undue reservation.

## Ethics Statement

The animal study was reviewed and approved by the animal Ethics Committee of the Third Xiangya Hospital, Central South University.

## Author Contributions

DL and HZ: conceptualization and formal analysis. DL, YL, and ZL: data curation, investigation, and methodology. DL and XY: funding acquisition. DL: project administration and writing – original draft. DL, HZ, and XY: resources and writing – review and editing. DL and YL: software. HZ and XY: supervision and validation. All authors contributed to the article and approved the submitted version.

## Conflict of Interest

The authors declare that the research was conducted in the absence of any commercial or financial relationships that could be construed as a potential conflict of interest.

## Publisher’s Note

All claims expressed in this article are solely those of the authors and do not necessarily represent those of their affiliated organizations, or those of the publisher, the editors and the reviewers. Any product that may be evaluated in this article, or claim that may be made by its manufacturer, is not guaranteed or endorsed by the publisher.

## Footnotes

1
http://www.ncbi.nlm.nih.gov/geo/

2
http://bioinfo.life.hust.edu.cn/hTFtarget#!/

3
https://string-db.org/cgi/input.pl?sessionId=LUwAMnwLiSDE&input_page_show_search=on

4
https://metascape.org/gp/index.html#/main/step1

5
http://gepia.cancer-pku.cn/

6
http://www.oncomir.org/

## Supplementary Material

Supplementary Figure 1 Gene expression profile across all tumor samples and paired normal tissues from Gene Expression Profiling Interactive Analysis (GEPIA). The height of the bar represents the median expression of certain tumor types or normal tissues. ACC, adrenocortical carcinoma; BLCA, bladder urothelial carcinoma; BRCA, breast invasive carcinoma; CESC, cervical squamous cell carcinoma and endocervical adenocarcinoma; CHOL, cholangiocarcinoma; COAD, colon adenocarcinoma; ESCA, esophageal carcinoma; GBM, Glioblastoma multiforme; HNSC, head and neck squamous cell carcinoma; KICH, kidney chromophobe; KIRC, kidney renal clear cell carcinoma; KIRP, kidney renal papillary cell carcinoma; LAML, Acute Myeloid Leukemia; LGG, brain lower grade glioma; LIHC, liver hepatocellular carcinoma; LUAD, lung adenocarcinoma; LUSC, lung squamous cell carcinoma; MESO, mesothelioma; OV, ovarian serous cystadenocarcinoma; PAAD, pancreatic adenocarcinoma; PCPG, pheochromocytoma and paraganglioma; PRAD, prostate adenocarcinoma; READ, rectal adenocarcinoma; SARC, sarcoma; SKCM, skin cutaneous melanoma; STAD, stomach adenocarcinoma; TGCT, testicular germ cell tumors; THCA, thyroid carcinoma; THYM, thymoma; UCEC, uterine corpus endometrial carcinoma; UCS, uterine carcinosarcoma.
