# Supplementary figures and images for: NR2F1-AS1 Promotes Pancreatic Ductal Adenocarcinoma Progression Through Competing Endogenous RNA Regulatory Network Constructed by Sponging miRNA-146a-5p/miRNA-877-5p
Source: Front Cell Dev Biol. 2021 Sep 28;9:736980. doi: 10.3389/fcell.2021.736980 (PMC8505696; doi:10.3389/fcell.2021.736980)

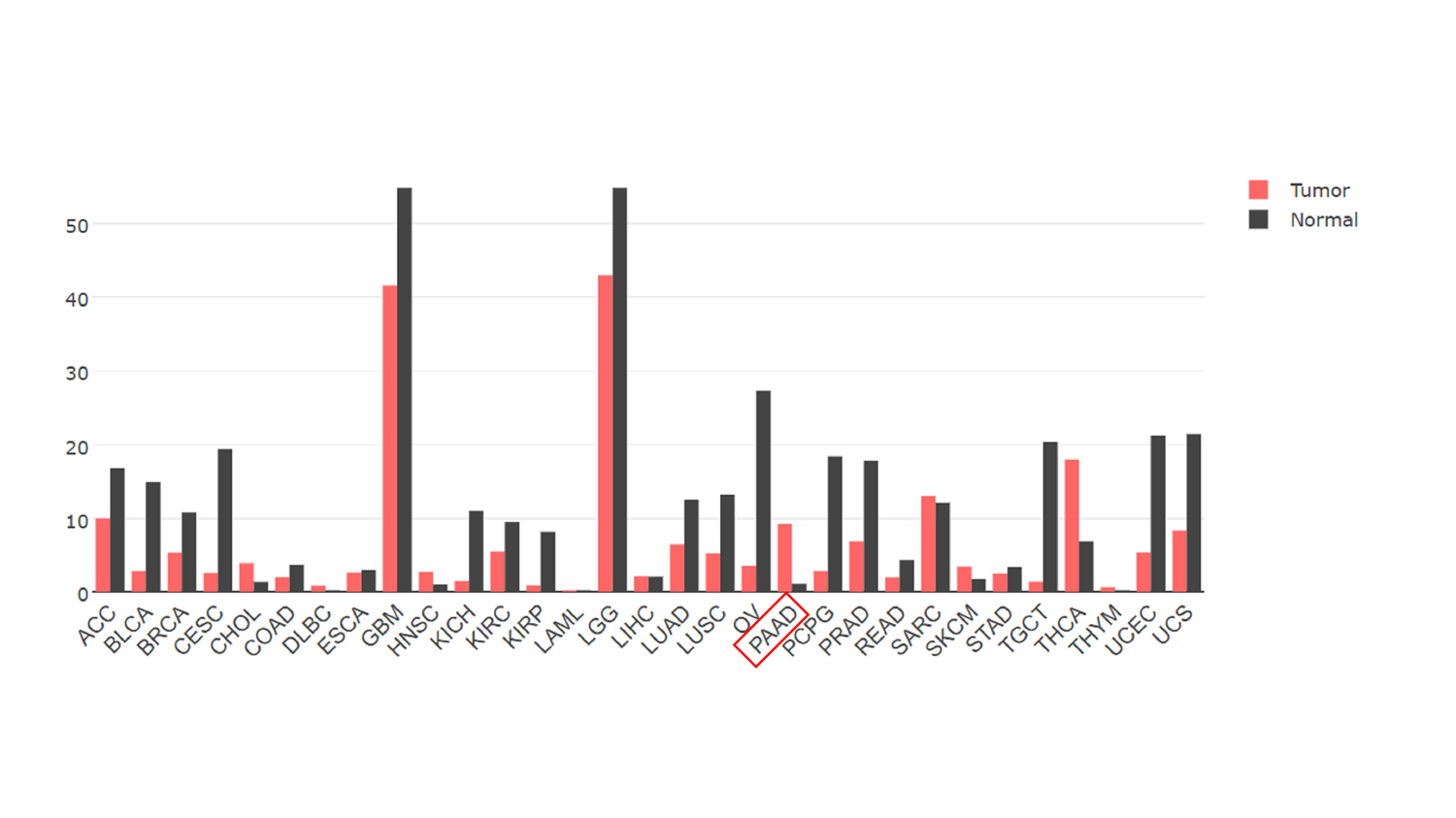

Supplement: Supplementary Figure 1 — Gene expression profile across all tumor samples and paired normal tissues from Gene Expression Profiling Interactive Analysis (GEPIA). The height of the bar represents the median expression of certain tumor types or normal tissues. ACC, adrenocortical carcinoma; BLCA, bladder urothelial carcinoma; BRCA, breast invasive carcinoma; CESC, cervical squamous cell carcinoma and endocervical adenocarcinoma; CHOL, cholangiocarcinoma; COAD, colon adenocarcinoma; ESCA, esophageal carcinoma; GBM, Glioblastoma multiforme; HNSC, head and neck squamous cell carcinoma; KICH, kidney chromophobe; KIRC, kidney renal clear cell carcinoma; KIRP, kidney renal papillary cell carcinoma; LAML, Acute Myeloid Leukemia; LGG, brain lower grade glioma; LIHC, liver hepatocellular carcinoma; LUAD, lung adenocarcinoma; LUSC, lung squamous cell carcinoma; MESO, mesothelioma; OV, ovarian serous cystadenocarcinoma; PAAD, pancreatic adenocarcinoma; PCPG, pheochromocytoma and paraganglioma; PRAD, prostate adenocarcinoma; READ, rectal adenocarcinoma; SARC, sarcoma; SKCM, skin cutaneous melanoma; STAD, stomach adenocarcinoma; TGCT, testicular germ cell tumors; THCA, thyroid carcinoma; THYM, thymoma; UCEC, uterine corpus endometrial carcinoma; UCS, uterine carcinosarcoma. [file Image_1.TIF]

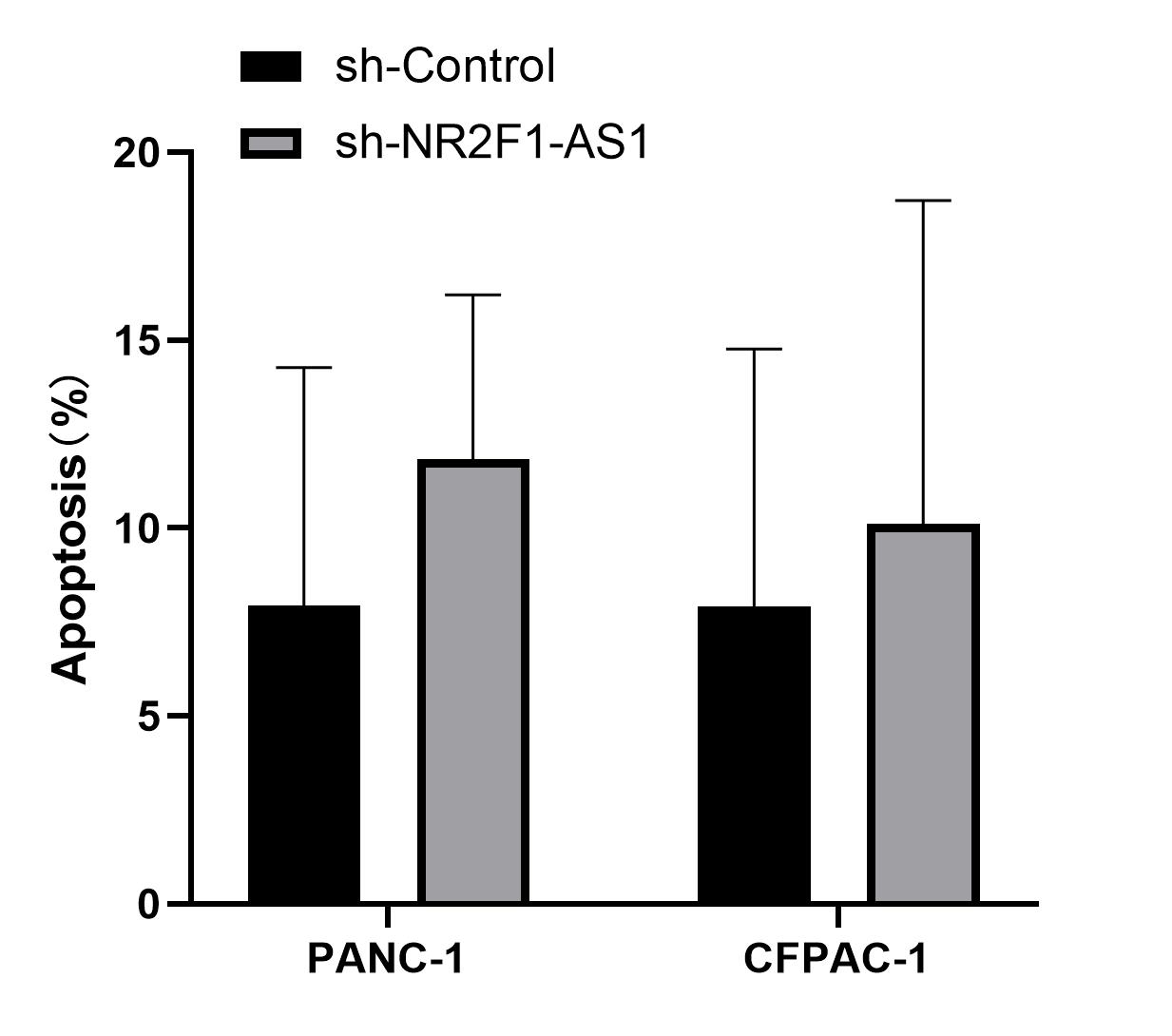

Supplement: Supplementary file 2 [file Image_2.JPEG]
